# Benzyl *N*-[(*Z*)-(1-methyl-2-sulfanyl­propyl­idene)amino]­carbamodithio­ate

**DOI:** 10.1107/S1600536812051008

**Published:** 2013-01-04

**Authors:** Mohammed Khaled bin Break, Sachin Mehta, M. Ibrahim M. Tahir, Karen A. Crouse, Teng-Jin Khoo

**Affiliations:** a School of Pharmacy, University of Nottingham Malaysia Campus, Selangor, Malaysia; b Department of Chemistry, Faculty of Science, Universiti Putra Malaysia, Malaysia

## Abstract

The title compound, C_12_H_16_N_2_S_3_, was obtained by the condensation reaction of *S*-benzyl dithio­carbazate and 3-mercaptobutan-2-one. The phenyl ring and thiol (SH) group are approximately perpendicular [S—C—C—C and N—C—C—S torsion angles = 67.8 (3) and 116.9 (2)°, respectively] to the rest of the mol­ecule. In the crystal, mol­ecules are linked by weak S—H⋯S and N—H⋯S hydrogen bonds, π–π inter­actions between the benzene rings [centroid–centroid distance = 3.823 (2) Å] and C—H⋯π inter­actions.

## Related literature

For biological applications of Schiff base ligands and complexes derived from *S*-benzyl­dithio­carbazate, see: Hossain *et al.* (1996 ▶); Tarafder *et al.* (2002 ▶). For related structures derived from *S*-benzyl­dithio­carbazate, which exhibit a similar geometry to the title compound, see: Khoo *et al.* (2005 ▶); How *et al.* (2007 ▶); Shan *et al.* (2011 ▶). For the synthesis, see: Tarafder *et al.* (2002 ▶).
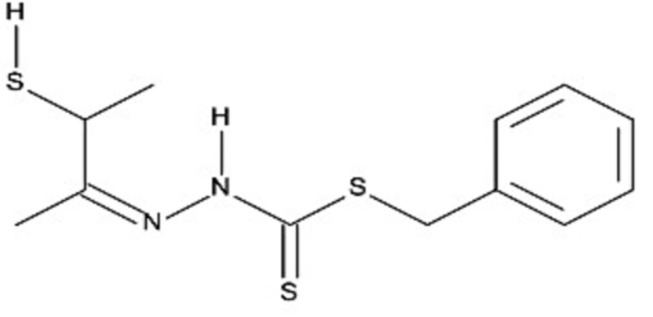


## Experimental

### 

#### Crystal data

C_12_H_16_N_2_S_3_
*M* *_r_* = 284.47
Monoclinic, 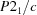

*a* = 16.3887 (4) Å
*b* = 8.3136 (2) Å
*c* = 10.1404 (3) Å
β = 90.234 (2)°
*V* = 1381.61 (6) Å^3^
*Z* = 4
Cu *K*α radiation
μ = 4.73 mm^−1^
*T* = 100 K
0.25 × 0.10 × 0.08 mm

#### Data collection

Oxford Diffraction Gemini diffractometer
Absorption correction: multi-scan (*CrysAlis PRO*; Agilent, 2011 ▶) *T*_min_ = 0.31, *T*_max_ = 0.68
7359 measured reflections
2615 independent reflections
2374 reflections with *I* > 2σ(*I*)
*R*_int_ = 0.027
Standard reflections: 0

#### Refinement

*R*[*F* ^2^ > 2σ(*F* ^2^)] = 0.044
*wR*(*F* ^2^) = 0.123
*S* = 0.99
2605 reflections
154 parameters
H-atom parameters constrained
Δρ_max_ = 0.55 e Å^−3^
Δρ_min_ = −0.60 e Å^−3^

### 

Data collection: *CrysAlis PRO* (Agilent, 2011 ▶); cell refinement: *CrysAlis PRO*; data reduction: *CrysAlis PRO*; program(s) used to solve structure: *SIR92* (Altomare *et al.*, 1994 ▶); program(s) used to refine structure: *CRYSTALS* (Betteridge *et al.*, 2003 ▶); molecular graphics: *Mercury* (Macrae *et al.*, 2006 ▶); software used to prepare material for publication: *publCIF* (Westrip, 2010 ▶).

## Supplementary Material

Crystal structure: contains datablock(s) global, I. DOI: 10.1107/S1600536812051008/nk2192sup1.cif

Structure factors: contains datablock(s) I. DOI: 10.1107/S1600536812051008/nk2192Isup2.hkl

Supplementary material file. DOI: 10.1107/S1600536812051008/nk2192Isup3.mol

Supplementary material file. DOI: 10.1107/S1600536812051008/nk2192Isup4.cml

Additional supplementary materials:  crystallographic information; 3D view; checkCIF report

## Figures and Tables

**Table 1:** Hydrogen-bond geometry (Å, °) *Cg*1 is the centroid of the C5–C10 ring.

*D*—H⋯*A*	*D*—H	H⋯*A*	*D*⋯*A*	*D*—H⋯*A*
S15—H151⋯S3^i^	1.38	2.96	4.186 (1)	146
N11—H111⋯S1^ii^	0.86	2.72	3.567 (2)	168
C7—H71⋯*Cg* ^iii^	0.95	2.97	3.827 (3)	152

Symmetry codes: (i) 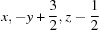; (ii) 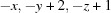; (iii) 
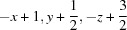
.

**Fig. 1.**
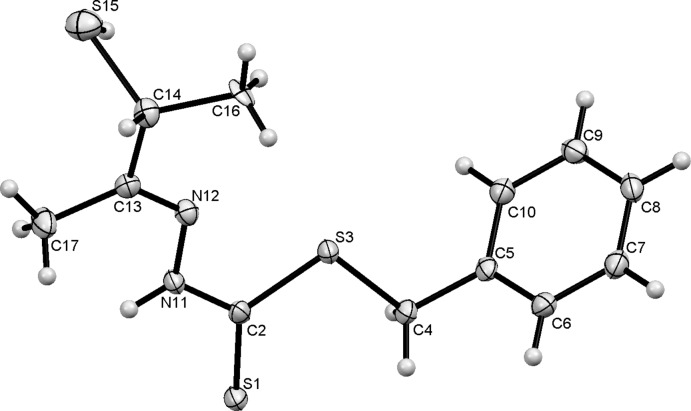
Molecular structure of the title compound (I) showing 50% probability displacement ellipsoids in addition to the atomic numbering scheme.

**Fig. 2.**
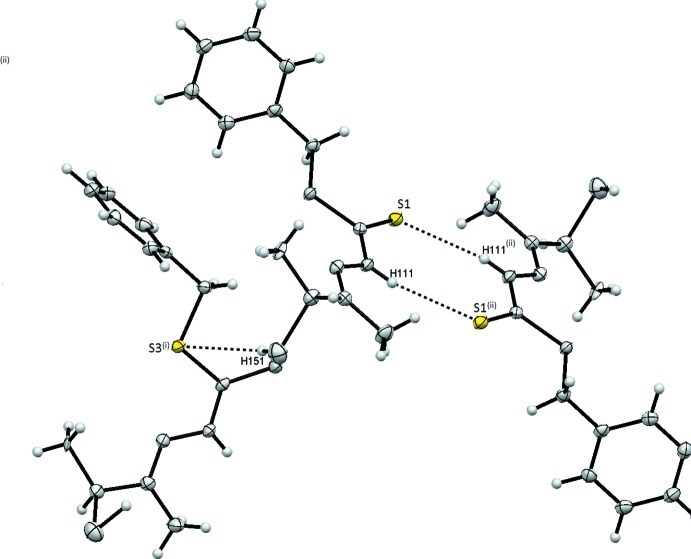
The molecules in the structure are stabilized by intermolecular H···S hydrogen bond interactions. Symmetry codes: (i)x, -y+3/2, z-1/2; (ii) -x, -y+2, -z+1

**Fig. 3.**
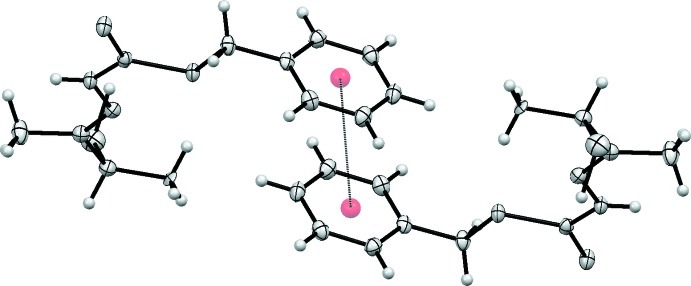
Molecules in the structure are linked by π- π interactions between pairs of benzene rings with centroid-centroid distance of 3.823 Å.

**Fig. 4.**
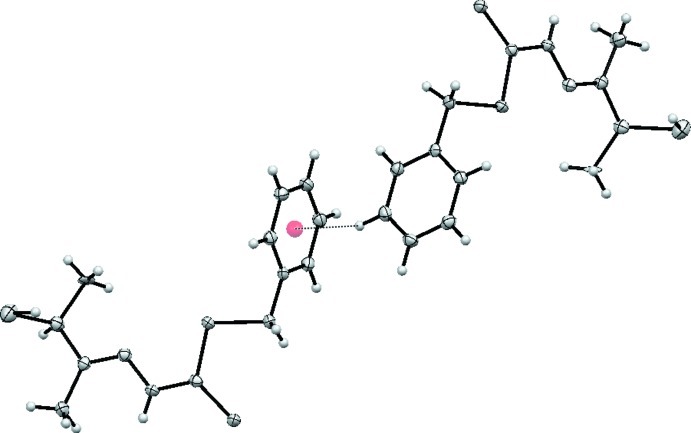
Diagram showing the C—H···π interactions between the molecules of the structure. Distance between the centroid of the benzene ring and the hydrogen atom of the neighbouring molecule is 2.970 Å.

## supplementary crystallographic information

### Comment

The past few years have seen a growing interest in the synthesis of Schiff base ligands and metal complexes specifically those derived from dithiocarbazates (Tarafder *et al.*, 2002; Hossain *et al.*, 1996). *S*-benzyldithiocarbazate (SBDTC) has been extensively studied due to the possibility of modifying its derivatives by the introduction of different substituents (Khoo *et al.*, 2005), furthermore, SBDTC-derived Schiff base ligands have been shown to possess antimicrobial and anticancer properties (Hossain *et al.*, 1996). Therefore, we have managed to synthesize the title compound, (I), which was a result of the condensation reaction between SBDTC and 3-mercaptobutan-2-one in order to investigate the bioactivity of this ligand and its metal complexes. In our course of research we have managed to grow crystals of the title compound, (I), from ethanol *via* the slow evaporation method.

X-ray crystallographic analysis has shown that the molecule [Fig.1] is planar with the phenyl ring and thiol group being nearly perpendicular to the rest of the molecule [S3—C4—C5—C10 and N12—C13—C14—S15 torsion angles of 67.8 (3)° and 116.9 (2)°, respectively]. The bond C2—N11 has a length of 1.3503 (3) Å whereas C13—N12 has a bond length of 1.278 (3) Å which is shorter than the former indicating that the latter possesses a double-bond character and belongs to the imine group. Similarly, the C2—S1 bond has a length of 1.659 (3) Å which is the shortest bond length relative to the other C—S bonds, and that indicates that it possesses a double bond character which further proves that the ligand exists in the thione tautomer in solid state. The bond lengths of the imine group (C=N) and that of the thione group (C=S) are similar to those reported in previously synthesized dithiocarbazate compounds [1.289 (3) Å for C=N, 1.664 (2) Å for C=S; Khoo *et al.*, 2005] and [1.285 (2) Å for C=N, 1.6667 (15) Å for C=S; Tarafder *et al.*, 2002], which indicates that such bond lengths are typical of Schiff base ligands derived from dithiocarbazates. The molecules in the crystal are linked together *via* intermolecular H···S [Fig.2] hydrogen bond interactions (Table 2). The benzene rings at (*x*, *y, z*) and (1 - *x*, 2 - *y*, 1 - *z*) are stacked parallel to each other and form π- π interactions with a separation of 3.823 Å and a shift distance of 1.539 Å [Fig.3.], while the distance between the planes of the benzene rings is 3.500 Å. Furthermore, there are C—H···π interactions (Table 2) between the molecules of the structure [Fig.4.] and the perpendicular distance between the plane of the benzene ring and H71 was found to be 2.790 Å. C*g* in (Table 2) refers to the centroid of the benzene ring present in the structure.

The molecule crystallizes in the conformer in which the thione sulfur is in a *trans* position with the ketone moiety across the C2—N11 bond but adopts a *cis* position with the phenyl group across the C2—S3 bond. The ketone moiety is *cis* to the phenyl group with respect to the C2—N11 bond. Such geometrical arrangements are similar to dithiocarbazate derived compounds reported previously (Khoo *et al.*, 2005; How *et al.*, 2007).

### Experimental

The method used for synthesis of the Schiff base ligand was a modified form of the one reported by (Tarafder *et al.*, 2002). (0.02) moles of *S*-benzyldithiocarbazate were dissolved in 40 ml absolute ethanol and then heated on a heating plate with constant stirring in order to ensure complete dissolving. Similarly, (0.02) moles of 3-mercaptobutan-2-one were mixed with 40 ml of absolute ethanol and heated on a heating plate for 10 minutes. The reactants were mixed and 2–4 drops of concentrated H_2_SO_4_ were added to the mixture. The mixture was kept on the heating plate for 5 more minutes and then cooled to 0°C in an ice-bath until the Schiff base precipitated. The Schiff base precipitated was filtered *via* suction filtration, washed with cold ethanol and dried over silica gel (yield 79.7%, m.p 361.45 K). Crystals suitable for X-ray analysis have been obtained *via* slow evaporation of ethanol over a period of 10 days.

### Refinement

The H atoms were all located in a difference map, but those attached to carbon atoms were repositioned geometrically. The H atoms were initially refined with soft restraints on the bond lengths and angles to regularize their geometry (C—H in the range 0.93–0.98 and N—H= 0.86 Å) and isotropic atomic displacement parameters (*U*_iso_(H) in the range 1.2–1.5 times *U*_eq_ of the parent atom), after which the positions were refined with riding constraints. H atom for the thiol group was located in a difference map and its coordinates were refined

### Crystal data

**Table:**

C_12_H_16_N_2_S_3_	*F*(000) = 600
*M**_r_* = 284.47	*D*_x_ = 1.368 Mg m^−^^3^
Monoclinic, *P*2_1_/*c*	Cu *K*α radiation, λ = 1.54180 Å
*a* = 16.3887 (4) Å	Cell parameters from 3777 reflections
*b* = 8.3136 (2) Å	θ = 4–71°
*c* = 10.1404 (3) Å	µ = 4.73 mm^−^^1^
β = 90.234 (2)°	*T* = 100 K
*V* = 1381.61 (6) Å^3^	Plate, yellow
*Z* = 4	0.25 × 0.10 × 0.08 mm

### Data collection

**Table:**

Oxford Diffraction Gemini diffractometer	2374 reflections with *I* > 2σ(*I*)
Graphite monochromator	*R*_int_ = 0.027
ω scans	θ_max_ = 71.4°, θ_min_ = 5.4°
Absorption correction: multi-scan (*CrysAlis PRO*; Agilent, 2011)	*h* = −19→20
*T*_min_ = 0.31, *T*_max_ = 0.68	*k* = −10→10
7359 measured reflections	*l* = −12→9
2615 independent reflections	

### Refinement

**Table:**

Refinement on *F*^2^	Primary atom site location: structure-invariant direct methods
Least-squares matrix: full	Hydrogen site location: difference Fourier map
*R*[*F*^2^ > 2σ(*F*^2^)] = 0.044	H-atom parameters constrained
*wR*(*F*^2^) = 0.123	Method = Modified Sheldrick *w* = 1/[σ^2^(*F*^2^) + ( 0.07*P*)^2^ + 2.47*P*], where *P* = (max(*F*_o_^2^,0) + 2*F*_c_^2^)/3
*S* = 0.99	(Δ/σ)_max_ = 0.001
2605 reflections	Δρ_max_ = 0.55 e Å^−^^3^
154 parameters	Δρ_min_ = −0.60 e Å^−^^3^
0 restraints	

### Special details

**Table:**

Experimental. The crystal was placed in the cold stream of an Oxford Cryosystems open-flow nitrogen cryostat with a nominal stability of 0.1 K.

### Fractional atomic coordinates and isotropic or equivalent isotropic displacement parameters (Å^2^)

**Table:**

	*x*	*y*	*z*	*U*_iso_*/*U*_eq_	
S1	0.09283 (4)	0.86651 (8)	0.60282 (6)	0.0199	
C2	0.14578 (15)	0.9210 (3)	0.4711 (3)	0.0161	
S3	0.25242 (4)	0.90367 (8)	0.45582 (6)	0.0169	
C4	0.28225 (15)	0.8290 (3)	0.6180 (2)	0.0188	
C5	0.37460 (15)	0.8307 (3)	0.6187 (2)	0.0169	
C6	0.41672 (16)	0.9417 (3)	0.6956 (3)	0.0206	
C7	0.50166 (17)	0.9473 (4)	0.6929 (3)	0.0225	
C8	0.54462 (16)	0.8430 (4)	0.6130 (3)	0.0219	
C9	0.50291 (16)	0.7324 (3)	0.5353 (3)	0.0214	
C10	0.41832 (16)	0.7260 (3)	0.5382 (3)	0.0197	
N11	0.11068 (12)	0.9875 (3)	0.3635 (2)	0.0170	
N12	0.16216 (13)	1.0374 (3)	0.2636 (2)	0.0175	
C13	0.13156 (16)	1.1118 (3)	0.1648 (3)	0.0177	
C14	0.19140 (16)	1.1704 (3)	0.0635 (3)	0.0204	
S15	0.17221 (5)	1.07040 (10)	−0.09485 (8)	0.0330	
C16	0.28359 (14)	1.1622 (3)	0.1108 (3)	0.0174	
C17	0.04289 (17)	1.1502 (4)	0.1460 (3)	0.0280	
H42	0.2606	0.8996	0.6857	0.0241*	
H41	0.2610	0.7201	0.6290	0.0236*	
H61	0.3876	1.0135	0.7489	0.0263*	
H71	0.5297	1.0237	0.7454	0.0292*	
H81	0.6020	0.8472	0.6120	0.0276*	
H91	0.5316	0.6627	0.4815	0.0271*	
H101	0.3910	0.6505	0.4850	0.0248*	
H141	0.1776	1.2846	0.0485	0.0260*	
H162	0.3131	1.2050	0.0476	0.0278*	
H161	0.2891	1.2182	0.1858	0.0279*	
H163	0.2963	1.0585	0.1218	0.0273*	
H171	0.0348	1.1957	0.0604	0.0430*	
H173	0.0259	1.2280	0.2111	0.0430*	
H172	0.0109	1.0536	0.1544	0.0427*	
H111	0.0595	1.0101	0.3634	0.0211*	
H151	0.1736	0.9172	−0.0413	0.0626*	

### Atomic displacement parameters (Å^2^)

**Table:**

	*U*^11^	*U*^22^	*U*^33^	*U*^12^	*U*^13^	*U*^23^
S1	0.0142 (3)	0.0288 (4)	0.0167 (3)	0.0005 (2)	0.0001 (2)	0.0022 (3)
C2	0.0139 (12)	0.0171 (12)	0.0173 (12)	−0.0001 (9)	−0.0009 (9)	−0.0038 (10)
S3	0.0123 (3)	0.0223 (3)	0.0159 (3)	0.0019 (2)	−0.0009 (2)	0.0016 (2)
C4	0.0160 (12)	0.0258 (14)	0.0146 (12)	0.0025 (10)	−0.0014 (9)	0.0029 (10)
C5	0.0155 (12)	0.0223 (13)	0.0130 (11)	0.0009 (10)	−0.0014 (9)	0.0051 (10)
C6	0.0218 (13)	0.0247 (14)	0.0154 (13)	0.0034 (11)	−0.0004 (10)	0.0000 (11)
C7	0.0212 (13)	0.0281 (15)	0.0182 (13)	−0.0022 (11)	−0.0040 (10)	0.0015 (11)
C8	0.0145 (12)	0.0294 (15)	0.0219 (13)	0.0000 (11)	−0.0016 (10)	0.0051 (11)
C9	0.0201 (13)	0.0242 (14)	0.0199 (13)	0.0043 (11)	0.0027 (10)	0.0021 (11)
C10	0.0196 (12)	0.0211 (13)	0.0183 (13)	−0.0012 (10)	−0.0021 (10)	0.0005 (10)
N11	0.0113 (9)	0.0217 (11)	0.0179 (11)	0.0009 (8)	−0.0003 (8)	0.0015 (9)
N12	0.0157 (10)	0.0176 (11)	0.0191 (11)	−0.0008 (8)	0.0001 (8)	0.0009 (9)
C13	0.0179 (13)	0.0184 (12)	0.0167 (12)	0.0003 (10)	0.0001 (10)	−0.0012 (10)
C14	0.0189 (13)	0.0195 (13)	0.0229 (13)	−0.0004 (10)	−0.0007 (10)	0.0036 (11)
S15	0.0372 (4)	0.0348 (4)	0.0269 (4)	0.0010 (3)	0.0047 (3)	0.0019 (3)
C16	0.0076 (11)	0.0178 (13)	0.0269 (14)	−0.0035 (9)	0.0069 (9)	0.0070 (10)
C17	0.0201 (14)	0.0412 (18)	0.0225 (14)	0.0051 (12)	−0.0010 (11)	0.0102 (13)

### Geometric parameters (Å, º)

**Table:**

S1—C2	1.659 (3)	C10—H101	0.941
C2—S3	1.761 (3)	N11—N12	1.384 (3)
C2—N11	1.350 (3)	N11—H111	0.859
S3—C4	1.823 (3)	N12—C13	1.278 (3)
C4—C5	1.514 (3)	C13—C14	1.504 (4)
C4—H42	0.972	C13—C17	1.499 (4)
C4—H41	0.976	C14—S15	1.834 (3)
C5—C6	1.390 (4)	C14—C16	1.585 (3)
C5—C10	1.393 (4)	C14—H141	0.988
C6—C7	1.393 (4)	S15—H151	1.385
C6—H61	0.937	C16—H162	0.880
C7—C8	1.382 (4)	C16—H161	0.896
C7—H71	0.947	C16—H163	0.894
C8—C9	1.389 (4)	C17—H171	0.955
C8—H81	0.941	C17—H173	0.966
C9—C10	1.388 (4)	C17—H172	0.963
C9—H91	0.926		
			
S1—C2—S3	124.83 (15)	C9—C10—H101	119.2
S1—C2—N11	122.70 (19)	C2—N11—N12	117.1 (2)
S3—C2—N11	112.46 (19)	C2—N11—H111	120.2
C2—S3—C4	102.18 (12)	N12—N11—H111	122.1
S3—C4—C5	105.39 (17)	N11—N12—C13	118.7 (2)
S3—C4—H42	109.5	N12—C13—C14	115.9 (2)
C5—C4—H42	111.0	N12—C13—C17	125.4 (2)
S3—C4—H41	108.9	C14—C13—C17	118.6 (2)
C5—C4—H41	111.4	C13—C14—S15	109.95 (18)
H42—C4—H41	110.4	C13—C14—C16	113.7 (2)
C4—C5—C6	120.2 (2)	S15—C14—C16	113.94 (19)
C4—C5—C10	120.5 (2)	C13—C14—H141	105.5
C6—C5—C10	119.2 (2)	S15—C14—H141	105.2
C5—C6—C7	120.4 (3)	C16—C14—H141	107.8
C5—C6—H61	119.6	C14—S15—H151	94.1
C7—C6—H61	120.1	C14—C16—H162	106.7
C6—C7—C8	120.1 (3)	C14—C16—H161	109.1
C6—C7—H71	119.7	H162—C16—H161	110.7
C8—C7—H71	120.2	C14—C16—H163	107.4
C7—C8—C9	119.8 (2)	H162—C16—H163	110.6
C7—C8—H81	119.6	H161—C16—H163	112.0
C9—C8—H81	120.6	C13—C17—H171	109.3
C8—C9—C10	120.2 (3)	C13—C17—H173	109.8
C8—C9—H91	120.0	H171—C17—H173	108.4
C10—C9—H91	119.8	C13—C17—H172	109.8
C5—C10—C9	120.3 (2)	H171—C17—H172	109.7
C5—C10—H101	120.5	H173—C17—H172	109.8

### Hydrogen-bond geometry (Å, º)

Cg1 is the centroid of the C5–C10 ring.

**Table:**

*D*—H···*A*	*D*—H	H···*A*	*D*···*A*	*D*—H···*A*
S15—H151···S3^i^	1.38	2.96	4.186 (1)	146
N11—H111···S1^ii^	0.86	2.72	3.567 (2)	168
C7—H71···*Cg*^iii^	0.95	2.97	3.827 (3)	152

Symmetry codes: (i) *x*, −*y*+3/2, *z*−1/2; (ii) −*x*, −*y*+2, −*z*+1; (iii) −*x*+1, *y*+1/2, −*z*+3/2.
